# Negotiating Access to Health and Wellbeing Support in Schools for Young People with Chronic Health Conditions in English Secondary Schools: A Qualitative Multi-Informant Study

**DOI:** 10.5334/cie.149

**Published:** 2025-02-17

**Authors:** Lauren Herlitz, Matthew Jay, Claire Powell, Ruth Gilbert, Ruth Blackburn

**Affiliations:** 1 NIHR Children and Families Policy Research Unit, Social Research Institute, IOE –UCL’s Faculty of Education and Society, UK; 2 NIHR Children and Families Policy Research Unit, UCL Great Ormond Street Institute of Child Health, UK

**Keywords:** chronic health, adolescents, school health, healthcare plans, inclusion, equality

## Abstract

**Background:**

Schools have a statutory duty to support pupils with medical conditions in England, but limited evidence exists on how support is managed in practice. This study explores young people’s, caregivers’, and school staff’s experiences of access to health and wellbeing support in state secondary schools for pupils with chronic health conditions.

**Method:**

We used an online qualitative survey design: one for young people or caregivers, and one for staff. Data was analysed using framework analysis, applying candidacy theory.

**Results:**

Twelve young people, 33 caregivers, and 18 secondary school staff responded to the survey. Participants described highly varied offers of health and wellbeing support, with caregivers and young people often unaware of what support schools could feasibly provide. Participants highlighted communication gaps and a lack of collaborative work between primary or secondary healthcare and schools. Many caregivers and young people reported that staff had insufficient understanding of their condition(s), had not trusted or believed them when they had explained their health needs, or had left them out of conversations about support. School staff also noted communication difficulties with caregivers. Many caregivers and staff described aspects of the secondary school setting that prohibited inclusivity including insufficient staff time, high pupil numbers, a focus on national attainment measures, and attendance targets.

**Conclusion:**

The support options that young people with chronic conditions can feasibly be offered should be clarified in government guidance. Further research is needed on the prevalence/utility of individual healthcare plans and on procedures to ensure that pupils with medical conditions are justly supported.

Around one in four children in England will have had a recorded chronic health condition by their 16^th^ birthday, according to hospital records (Jay et al., 2024). Chronic health conditions are physical, physiological, neurological and/or mental health problems that are enduring and have an impact on everyday activities (Spencer, Wright, et al., 2023). Evidence reviews have found that children and young people with chronic conditions experience higher school absence, have difficult school experiences (e.g., experiencing exclusion from school activities, bullying) and lower academic attainment more frequently than their peers (Jay et al., 2023; Lum et al., 2017; Spencer, Hugh-Jones, et al., 2023).

Schools have a statutory duty to support pupils with medical conditions to ensure that they remain healthy and safe at school, can fully access education, and are not disadvantaged compared to their peers (Children and Families Act 2014 c.6 Part 5; Equality Act 2010 c.15 Part 6; Department for Education, 2015). Government guidance highlights the diversity of conditions that pupils may experience and the need for multiple parties to work together (e.g., local authorities, health professionals, and where appropriate, social care professionals), noting that: “*Governing bodies should therefore ensure that the focus is on the needs of each individual child,”* (Department for Education, 2015, p.7).

Government guidance recommends that medical conditions policies include: responsibility for staff training; plans to ensure staff are aware of pupils with medical conditions and their support needs; processes for monitoring pupils’ Individual Healthcare Plans (IHPs) – a non-statutory document that a school may create to summarise a pupils’ needs and set out actions/expectations of different parties (Department for Education, 2015). However, a survey of 117 schools found only 23 schools (20%) had a medical conditions policy that followed statutory guidelines; many schools had no policy at all or a policy of poor quality (Health Conditions in Schools Alliance [HCSA], 2017). Surveys of caregivers, teachers and local authority staff have found that many pupils with medical conditions have no IHP (Creighton, 2012; Dyson et al., 2008; Jones et al., 2021), and data on IHPs are not routinely captured in the government’s National Pupil Database, indicating an evidence gap on how schools manage and co-ordinate support for pupils with chronic health conditions.

While school governing bodies are responsible for developing and ensuring the implementation of policies for pupils with medical conditions, any school staff member may be involved in the care of a pupil with a medical condition (Department for Education, 2015). Although school nurses have the skills to support pupils with chronic conditions, high workforce shortages and workload demands in England mean that most nurses work across many schools and spend little time directly working with pupils (Booth, 2015; Buchan, 2020; Children’s Commissioner, 2016). School leaders, teaching staff, pastoral care staff, and Special Educational Needs Co-ordinators (SENCOs) may provide support, depending on individual pupils’ needs and whether they have a close, trusted relationship with a particular staff member (Spencer, Wright, et al., 2023). Depending on their condition, specialist health professionals may support pupils’ engagement in education, and they may receive ongoing support from their general practitioner. Local authority staff may organise care for pupils with medical needs who cannot attend school and allocate funding for an Education Health and Care Plan (EHCP) for pupils with significant special education needs and/or social care needs (Department for Education, 2015, 2023). In short, many people may potentially be involved in supporting pupils with medical conditions but there is little evidence about how care at school is managed in practice.

Government policies and research highlight that parents and carers (hereafter referred to as ‘caregivers’) have a key role in communicating with the school about their child’s medical needs and contributing to healthcare plans, monitoring their child’s health, and liaising with professionals (Bowtell et al., 2018; Department for Education, 2015; Kelada et al., 2021). In multiple studies, educators and caregivers have reported poor communication between families, schools and health services (Barlow et al., 1998; Hinton & Kirk, 2015; McLoone et al., 2011). A qualitative study in Australia also found caregivers’ experiences differed depending on the condition, including whether specialist health professionals liaised with schools or school staff collaborated with caregivers (Bowtell et al., 2018).

This study aims to contribute evidence on the experience of accessing health and wellbeing support in schools in England, examining commonalities across young people with a wide range of different conditions. We adopted the conceptual model of access proposed by candidacy theory (Dixon-Woods et al., 2006), originally developed to examine healthcare access, which proposes that both services and the people that use them are continually establishing and defining what problems are worthy of attention and intervention. It asserts that accomplishing access requires individuals to perceive themselves as an appropriate candidate for a service and carry out work to negotiate access; the amount, difficulty, and complexity of that work can be a barrier to receiving care (Dixon-Woods et al., 2006). Existing evidence on the experiences of pupils with chronic health conditions and their caregivers has shown that staff support is affected by their perception and understanding of the condition, suggesting the relevance of candidacy theory in this context (Bowtell et al., 2018; Dyson et al., 2010; Logan et al., 2007). Candidacy theory has previously been used to explain healthcare access in underserved youth populations (Herlitz, Ashford, Baldwin, et al., 2024; Nkosi et al., 2019; Normansell et al., 2016), and to explore school staff and parents’ experiences of introducing a primary health nurse into a school setting (Dennis et al., 2016).

We asked: what were young people’s, caregivers’ and school staff’s experiences of accessing health and wellbeing support in state secondary schools for pupils with chronic health conditions?

## Method

### Design

We used a qualitative survey design (Braun et al., 2021) to explore experiences of school support, creating two online surveys: one for young people, parents or carers (hereafter referred to as caregivers), and one for school staff (see registered protocol https://osf.io/b6ysr). The study was approved by the UCL Research Ethics Committee (REC reference: 17893/005). We have included additional methodological details (e.g. definitions used and consultation with young people and caregivers) in supplementary file 1.

### Sampling and Recruitment

Table 1 presents participants’ eligibility criteria. Using convenience sampling, we recruited young people and caregivers by advertising the study through relevant networks, using emails and social media, and directly approached a broad range of health, mental health and educational organisations working with young people (see supplementary file 2). We recruited school staff through the Health Conditions in Schools Alliance, Anna Freud School in Mind, UCL Great Ormond Street Institute of Child Health, the National Education Union (NEU), the UCL Institute of Education secondary school student teachers’ network and our professional networks.

**Table 1:** Participant inclusion criteria.


TYPE OF PARTICIPANT	INCLUSION CRITERIA

Young people	Aged 16–25 years Had one or more chronic health condition(s) while at secondary school. Attended a mainstream secondary school in England.

Caregivers	Cared for a young person aged 11–25 years. Young person had one or more chronic health condition(s) while at secondary school. Young person attended a mainstream secondary school in England.

School staff	Worked in a mainstream secondary school in England. Had one of the following roles: Teaching or supporting students in the classroom, e.g., classroom teacher, teaching assistant, SENCO, without a leadership role. A school leader with responsibility for writing school policies, e.g., head of year, head of department, deputy head, headteacher. Educational psychologist


### Data Collection

We used Qualtrics (Qualtrics, Provo, UT) as the online platform. Interested individuals followed a link to the survey landing page, which introduced the study and gave a link to the information sheet. The consent form followed and if participants responded ‘yes’ to all questions, they could progress to start the survey. Informed consent to participate was obtained from all study participants.

The young people and caregiver survey was live from 7^th^ November 2022 to 31^st^ January 2023, filtering the questions for each type of participant. Participants were asked open-ended questions about the child or young person’s condition(s) and school experiences as well as several demographic questions (see supplementary file 3). We piloted the survey with a young person from a mental health charity participation group to make the questions clear and understandable. The school staff survey was live from 30^th^ January to 5^th^ May 2023. Participants were asked open-ended questions about how pupils with chronic health conditions were supported, demographic questions, and questions about their role and teaching experience (see supplementary file 3). We piloted the school staff survey with two teachers to ensure the questions were understandable. At each survey’s end, participants could enter a prize draw for a £50 voucher. On average, young people completed the survey in 24 mins, caregivers in 38 mins and school staff in 23 mins.

### Data Analysis

We described the distribution of demographic characteristics of those participating, including missing characteristics. LH conducted thematic analysis of the data from open-ended questions (Miles & Huberman, 1994) using NVivo 12 software (Lumivero, 2017). LH read and re-read the data and conducted inductive line-by-line coding. LH applied candidacy theory as a theoretical framework to enhance the explanatory power of the analysis (Anfara & Mertz, 2015). LH translated the theory’s seven features of candidacy into the context of the study (see Table 2) and applied the features as an initial way of grouping the codes into higher-order themes. LH then re-checked the content of each code and its meaning, re-coding and re-grouping where necessary, until the final themes were constructed. LH discussed theme development with the wider team, and after iterations, the team agreed on the final themes.

**Table 2:** Description of Candidacy Theory, adapted from Dixon-Woods et al (2006).


CANDIDACY FEATURE	DESCRIPTION OF FEATURE

Identification of candidacy	Whether/how young people or caregivers recognise a health condition as needing support from school.

Navigation of services	Young people and caregivers’ awareness of the support available and how to get it put in place.

Permeability of services	The ease with which young people and caregivers can use support services, e.g., whether referrals are required, whether there are waiting lists.

Appearing at services	Whether young people and caregivers can clearly explain their/their child’s needs and appear credible.

Adjudication by educators and other professionals	How professional judgements about a child’s needs affect their access to support.

Offers of, resistance to, services	Whether young people or caregivers resist offers of support.

Operating conditions and local production of candidacy	Aspects of the school setting that affect interactions between educators, caregivers, and pupils with health needs.


## Results

We present the participants’ characteristics followed by the key themes that we constructed. In quotes where young people have multiple conditions, to preserve anonymity, we have named the first condition and indicated ‘other conditions’.

### Sample Characteristics

Tables 3 and 4 present participants’ characteristics. There was a notably high degree of missingness for gender among young people and caregivers, with 25% missing for young people and 61% missing for caregivers, though no missingness for school staff. 12 of the 19 young people who had filled out the consent form completed the survey, and 33 of the 46 caregivers who had filled out the consent form completed the survey. Young people and caregivers were from diverse locations across England. Two-thirds of both young people and caregivers reported that they or their child had more than one condition. A wide range of different conditions (49 in total) affecting different body systems were reported across the young people and caregiver sample (N = 45) (see supplementary file 4).

**Table 3:** Young people and caregiver demographic characteristics.


DEMOGRAPHIC CHARACTERISTICS		YOUNG PEOPLEN (%)	CAREGIVERN (%)

Gender	Female	6 (75)	12 (92)

	Male	2 (25)	1 (8)

	No response	4 (33)	20 (61)

	Total	12 (100)	33 (100)

Age	16–18 years	5 (42)	n/a

	19–25 years	7 (58)	n/a

	26–35 years	n/a	0 (0)

	36–45 years	n/a	7 (22)

	46–55 years	n/a	20 (63)

	56–65 years	n/a	5 (16)

	No response	n/a	1 (3)

	Total	12 (100)	33 (100)

Ethnicity	White British	8 (67)	24 (73)

	White European or Other	1 (8)	4 (12)

	Black British, Caribbean, or African	0 (0)	2 (6)

	Asian or Asian British	2 (17)	0 (0)

	Mixed or multiple ethnic group	0 (0)	0 (0)

	Other ethnic group	0 (0)	1 (3)

	Prefer not to say	1 (8)	2 (6)

	Total	12 (100)	33 (100)

Location	North England	1 (8)	6 (19)

	Yorkshire and the Humber	4 (33)	6 (19)

	Midlands	1 (8)	4 (13)

	East of England	1 (8)	2 (6)

	London	1 (8)	7 (22)

	South England	4 (33)	7 (22)

	No response	n/a	1 (3)

	Total	12 (100)	33 (100)

Presence of Individual Health Plan	Yes	9 (82)	17 (52)

	No	2 (18)	16 (48)

	No response	1 (8)	n/a

	Total	12 (100)	33 (100)

Presence of Special Educational	Yes	7 (58)	18 (52)

	No	5 (42)	16 (48)

	No response	n/a	1 (3)

	Total	12 (100)	33 (100)

Number of conditions	Single condition	4 (33)	11 (33)

	Two conditions	4 (33)	9 (27)

	Three conditions	2 (17)	5 (15)

	Four or more conditions	2 (17)	8 (24)

	Total	12 (100)	33 (100)


**Table 4:** School staff demographic characteristics.


DEMOGRAPHIC CHARACTERISTICS		SCHOOL STAFF N (%)

Gender	Female	16 (89)

	Male	2 (11)

	Total	18 (100)

Age	19–25 years	1 (6)

	26 to 35 years	4 (22)

	36 to 45 years	5 (28)

	46–55 years	6 (33)

	56–65 years	2 (11)

	Total	18 (100)

Ethnicity	White British	15 (83)

	White European or Other	1 (6)

	Black British, Caribbean, or African	0 (0)

	Asian or Asian British	1 (6)

	Mixed or multiple ethnic group	1 (6)

	Other ethnic group	0 (0)

	Prefer not to say	0 (0)

	Total	18 (100)

Location	North England	0 (0)

	Yorkshire and the Humber	0 (0)

	Midlands	6 (33)

	East of England	3 (17)

	London	0 (0)

	South England	9 (50)

	Total	18 (100)

Role in school	Staff teaching or supporting students	6 (33)

	Staff with a leadership role	10 (56)

	Educational psychologist	2 (11)

	Total	18 (100)

Length of time teaching	Under 3 years	0 (0)

	3–5 years	2 (11)

	6–9 years	4 (22)

	10 years or more	12 (67)

	Total	18 (100)

Size of school population	Less than 500 students	5 (29)

	500 to 899 students	2 (12)

	900 to 1199 students	3 (18)

	1200 or more students	7 (41)

	No response	1 (6)

	Total	18 (100)


Eighteen of the 35 school participants who had filled in the consent form completed the survey. Most school staff participants were based in South England or the Midlands. Just over half were school leaders and a third were teachers or teaching support staff (see Table 4). Most had been teaching for over 10 years.

### Views and Experiences of Accessing Health and Wellbeing Support in School

We constructed four themes on young people’s, caregivers’, and school staff’s perspectives on accessing health and wellbeing support (see Table 5). Our data aligned with four of the seven features of candidacy: navigation (theme 1), permeability (theme 2), adjudication (theme 3) and operating conditions and local production of candidacy (theme 4).

**Table 5:** Key themes of views and experiences of accessing health and wellbeing support.


Theme 1	Navigating What Support is Available: “Being asked what would help to be told it can’t be done”

Theme 2	The Permeability of Health and Wellbeing Support: “You don’t know what is happening and you feel unable… to make direct contact”

Theme 3	The Adjudication of Health and Wellbeing Support by Staff: “I wish that school had listened to me”

Theme 4	Aspects of the School Setting and Institutional Context were Barriers to High Quality Support: “The whole system is based on minimising the inconvenience, rather than embracing their differences” Sub-themes: Pressures on Staff Time and School Resources: “Teaching staff having no time to get to know and understand my son” Providing Flexible Learning versus National Measures of Attainment: “The balance between supporting the condition and pupil and allowing the other pupils to be able to learn uninterrupted” Attendance Targets and How Attendance Policies are Communicated: “The awful attendance awards which they can never achieve”


**Theme 1: Navigating What Support is Available: “Being asked what would help to be told it can’t be done”**. Participants described highly varied offers of health and wellbeing support. There was an overarching sense that navigating support was largely a random and piecemeal process. Support reported included healthcare plans, reasonable adjustments (see supplementary file 5), support from staff, support from health professionals external to the school, and access to alternative provision (see Table 6). However, caregivers and young people were often not aware of what was possible or viable for the school to put in place. One caregiver suggested there should be:

“*Some sort of generic passport that offers options that school can and will deliver, that all staff are familiar with and you can pick and choose what would work for your child so you are not being asked what would help to then be told it can’t be done or won’t be done even if it is agreed*.” Caregiver AB of young person with hemiplegia and another condition

**Table 6:** Participants’ perspectives on navigating different types of health and wellbeing support.


TYPE OF SUPPORT	KEY FINDINGS

Healthcare plans	Several staff, caregivers and one young person positively described accessing healthcare plans as a mechanism for considering a child’s need holistically and enhancing communication between relevant parties. Many caregivers reported difficulties they had accessing an EHCP: knowing their rights in being able to apply for an EHCP or the criteria for getting one knowing how to communicate with the local authority, particularly for rarer conditions/those harder to diagnose finding that professionals did not know how to write letters that effectively communicated pupil’s needs

Reasonable adjustments	Participants reported many kinds of adjustments to prevent pupils from being at a disadvantage compared with their peers (see appendix 5). The most often reported were exam support and adjustments to help manage the duration of the school day (e.g., access to a pastoral room for respite, or having a bespoke or reduced timetable). Several caregivers and young people reported desired adjustments that they had not received, but which other participants had been given (e.g., a bespoke timetable, flexibility on staying seated in class, exam support).

Support from staff within the school	Many participants recounted support from a particular staff member, often the SENCO or pastoral team, but also from Heads of Year, teaching assistants, the school nurse, teachers, and mental health leads. The nature of the support varied, including: acting as a key worker with oversight of a pupil’s care providing additional tuition or classroom support providing emotional support referring to other services helping a pupil to access exam support or an EHCP Many caregivers and some staff and young people highlighted the limited nature of support: lack of a key worker low availability of teaching assistants or school nurses a need for more mental health support in school insufficient whole-school support

Support from health professionals external to the school	Several caregivers and staff, and a young person reported receiving help from condition-specific health professionals who liaised with school (e.g., asthma nurse) or from a local authority medical needs team. Two caregivers noted that they would have liked easier contact with medical professionals within the school setting.

Access to education outside of mainstream school	Several caregivers and staff reported that pupils had transitioned to and from mainstream school to other forms of provision as their health status had changed. For some caregivers, alternative provision was paid for by the local authority, while others had funded private tuition themselves when there appeared to be no other option. Several caregivers had wished that they had been given information about alternatives to state and private schools, concerned that mainstream schooling had worsened their child’s health.


**Theme 2: The Permeability of Health and Wellbeing Support: “You don’t know what is happening and you feel unable… to make direct contact”**. Participants reported that effective health and wellbeing support relied upon good communication between health and education services, young people and caregivers. Many participants highlighted gaps in communication between primary or secondary care and schools, or a lack of “joined-up thinking” (Caregiver B). They reported instances of being unable to get through to a named professional easily or receive information that they needed quickly:

“*External agencies don’t get in touch with schools especially well quite often, parental contact is a struggle very often, even getting a doctors letter for exams access arrangements takes months.”* School leader

Consequently, accessing support could be burdensome for caregivers and inefficient for all parties. One staff member, quoted in the theme’s title, noted that support structures within the school itself could be impermeable, for example, a teacher being prevented from becoming involved in home-school liaison if the role was assigned to another staff member. Several school staff highlighted the difficulty of effectively supporting pupils that did not qualify or were waiting lengthy periods for help from Child and Adolescent Mental Health Services (CAMHS), where there were high thresholds and long waiting lists.

**Theme 3: The Adjudication of Health and Wellbeing Support by Staff: “I wish that school had listened to me”**. Even if caregivers and young people were aware of the support they needed, professional judgements from school staff about the support pupils were entitled to or would benefit from affected pupils’ access to support. Many caregivers and staff, and a few young people gave a positive example of a staff member caring for pupils, characterised by empathy and understanding, and staff engaging caregivers and pupils in regular and responsive conversations about a pupil’s needs.

*“This person was authoritative but empathetic. She never conveyed to our son that she was confused or frustrated by his behaviour. She had high expectations but knew when to pull back. She was brilliant.” Caregiver C of child with anxiety and another condition*.

Unfortunately, most caregivers and young people highlighted that staff had insufficient understanding of their health condition, several caregivers and staff described a lack of staff knowledge of special educational needs, and many school staff noted gaps in their knowledge on supporting pupils with poor mental health. Several caregivers and young people believed that staff had simply been disinterested in their health and wellbeing.

“*I wasn’t as important as other kids because I missed school and I needed extra thought and planning*.” Young person with a disease of the digestive system

Many caregivers and some young people reported that school staff had not trusted them or believed them when they had explained their health needs; some pupils had been accused of lying about their condition and some caregivers (including the caregiver quoted in the theme’s title) had been referred to the safeguarding team or threatened with fines or de-registration. A lot of caregivers and young people said that they had been ignored or left out of conversations about their/their child’s health and education support.

“*My son had a key worker who was fabulous. The school moved her responsibilities without telling us*.” Caregiver_A of a child with sickle cell anaemia.

Several caregivers and a young person described accessing support as a ‘*fight’* or a ‘*battle’*, while many school staff also reported that it could be difficult to get caregivers to communicate with them or work together as a team.

**Theme 4: Aspects of the School Setting and Institutional Context were Barriers to High Quality Support: “The whole system is based on minimising the inconvenience, rather than embracing their differences”**. Many caregivers and staff described aspects of the secondary school setting and institutional context that prohibited schools from being able to accommodate the individual needs of pupils with chronic health conditions, as conveyed by a caregiver in the theme’s title. We constructed three sub-themes: 1) pressures on staff time and school resources; 2) providing flexible learning versus national measures of attainment; 3) the harmful impact of attendance targets and how attendance policies are communicated.

***Pressures on Staff Time and School Resources: “Teaching staff having no time to get to know and understand my son”***. Many caregivers and staff reported a lack of staff time, high pupil numbers, and/or a shortage of staff meant it was difficult to address a pupil’s individual needs, to get to know a pupil well, or read or digest important information on the healthcare plan. Staff noted that they also did not have the time they would need to adapt lesson resources for home learning or help an individual pupil catch-up. Secondary schools were large organisations, with many staff and frequent staff changes, and participants reported it was difficult to communicate an individual pupil’s needs to every teacher.

“*[Start of] secondary school, a large number of temporary teaching and office staff in addition to different teachers for different subjects. Some teachers had not read IHP; old IHP resurfaced several times which was irrelevant*.” Caregiver M of child with type 1 diabetes.

Several school staff highlighted that there were constraints on physical space in schools so there may not be a separate room available for respite, one-to-one or small group support, and some classrooms were not suitable for wheelchairs. Caregivers also reported budget constraints to offering tailored care, for example, in terms of staffing or providing suitable adjustments.

***Providing Flexible Learning versus National Measures of Attainment: “The balance between supporting the condition and pupil and allowing the other pupils to be able to learn uninterrupted”.*** Multiple caregivers said that their children had needed a more ‘*flexible’* approach to learning and assessment. This encompassed: tailored responses to absence (e.g., offering online learning, gradual re-integration into school, additional tuition); offering a broad curriculum that encompassed practical skills, project/independent work, and craftsmanship; and flexibility around assessment (e.g., extensions to homework or coursework, grading based on project work). However, many caregivers and several staff reported that achieving attainment targets were prioritised by schools over the quality of care for pupils with chronic health conditions:

“*I found the SENCO team very unhelpful as they were only really interested in her academic levels and because she was average and hitting basic targets, they were not interested.”* Caregiver_AB of young person with hemiplegia and another condition

***Attendance Targets and How Attendance Policies are Communicated: “The awful attendance awards which they can never achieve”***. Many caregivers thought that attendance targets, with related attendance awards and penalties, unfairly depreciate and punish vulnerable pupils (and their caregivers) who could not avoid health-related absences. They reported that targets and awards could create a pressure to attend even when it would risk a deterioration in a pupil’s health (also reported by one staff member), isolate pupils from their peers if class awards were given for whole-class attendance, and negatively affect a pupil’s self-esteem:

“*… made to feel a failure for being unwell*” Caregiver E of a child with a heart condition and other conditions.

“*The awful attendance awards which they can never achieve because they need to be responsible in managing their condition*” Caregiver N of a child with depression and other conditions.

Several caregivers thought that attendance penalties and awards could reinforce a punitive school response to absence rather than seeing absence as flag for health and wellbeing needs and focus attention on attendance and away from a broader view of a pupil’s education (regardless of setting). Some caregivers noted that as well as schools communicating fines and threats of custodial sentences for non-attendance, promoting a message that a pupil would not achieve academic qualifications or be employable if they did not attend could alienate and depreciate pupils with chronic health conditions.

## Discussion

Health and wellbeing support in secondary schools for pupils with chronic health conditions was difficult to access, it was unclear what support was possible or feasible to put in place, and support needs were poorly communicated across school staff community, based on young people and caregivers’ reports. Collaboration between schools and healthcare professionals was often challenging or non-existent, with the burden often falling upon caregivers to advocate for their child in one or both settings. According to participants’ accounts, pressures on staff time and resources, and a school-level focus on national attainment measures and attendance targets were barriers to young people with chronic health conditions receiving high quality support that met their individual needs. In short, the current system of support for these pupils was highly variable and often poor.

Caregivers and young people in other qualitative studies have also identified concerns about negotiating access to support when their child has returned to school following serious illness (Beeler et al., 2021; Finlay-Jones et al., 2023; Hopwood et al., 2024; Paré-Blagoev et al., 2019). Existing evidence shows that communication between caregivers, schools and health services are often inadequate (Paré-Blagoev et al., 2019; Uhm et al., 2020; Vanclooster et al., 2018). The consequences of poor support for pupils with chronic health conditions are severe, with poorer educational outcomes and worse mental health (Hopwood et al., 2024; Jay et al., 2023).

### Implications for Policy and Practice

Young people with chronic health conditions need support to participate in school, and schools are legally obliged to ensure that these pupils have the same opportunities to access education as their healthier peers (Department for Education, 2015; Hopwood et al., 2024; Spencer, Hugh-Jones, et al., 2023). However, this study shows that in practice, the experience of accessing support is difficult, demotivating, and encourages disengagement from education.

Participants’ accounts suggest that government guidance on supporting pupils with medical conditions should be updated to provide a clear account of schools’ health and wellbeing support options. Following this guidance, schools can clarify their own support offer so that caregivers and young people can constructively discuss choices from a feasible list of what could be offered. While one would expect variation in support due to differences among medical conditions, the experiences of fatigue and falling behind from absence are common among most pupils with chronic conditions (Spencer, Wright, et al., 2023). Consequently, we suggest that adjustments related to managing the duration of the school day, resources dedicated to catching up after absence, and support with assessments, should be a part of every discussion after persistent health-related absence. Caregivers reported that a named individual responsible for overseeing care was important, and caregivers and staff in multiple other studies have suggested that a school liaison officer (based in education or healthcare services) can improve communication among all parties, identify training needs, and monitor pupil’s progress (Holmström et al., 2018; McGlynn et al., 2023; Vanclooster et al., 2018).

Schools are under pressure to ensure high attendance after elevated absence levels after the COVID-19 pandemic (Department for Education & The Rt Hon Gillian Keegan MP, 2024). Government guidance advises schools not to penalise pupils with medical conditions for health-related absences and highlights the importance of listening to caregivers and pupils (Department for Education, 2015). Guidance says, “where used sensitively and without discrimination” (p.10), schools can praise and reward improvements in attendance (Department for Education, 2022). However, participants in our study, and others, have highlighted that attendance awards are, in their nature, discriminatory as young people with chronic health conditions cannot achieve 100% attendance, and attendance is not within their control (Hopwood et al., 2024). Caregivers reported that awards and fines are communicated bluntly by schools and are not balanced with offers of support for those with additional needs. We recommend that schools and local authorities positively communicate their intention to listen to pupils and families and understand their needs when a pupil is persistently absent from school and to offer support. We recommend that pupils absent from school for medical conditions are recognised for the challenges that they face and their efforts to engage with learning whether at home, at hospital or at school, and that schools carefully consider the balance of benefits and potential harms for awards given to pupils solely for attendance.

### Implications for Future Research

Several participants noted that IHPs had been a useful tool for improving communication between caregivers and young people with chronic health conditions, schools, and other relevant parties, and IHPs are promoted as good practice in government guidance and by healthcare charities (Department for Education, 2015; HCSA, n.d.). Currently there are no studies or routinely collected data to assess the usage of IHPs in schools. Further research on the prevalence and utility of IHPs is needed, with recommendations for whether the presence of an IHP should be recorded in the National Pupil Database.

Several caregivers reported that they had to ‘fight’ to ensure that their child’s educational and health needs were met, a finding supported by other studies (Beeler et al., 2021; Paré-Blagoev et al., 2019). Not all children will have a caregiver that is able to advocate for their needs, which can increase inequity in accessing support (Herlitz, Ashford, Powell, et al., 2024). Multiple reviews have found that parents of children with chronic health conditions experience poorer mental health, higher parental stress, and higher demands on their time, for example, from carrying out healthcare-related tasks (Cohn et al., 2020; Cousino & Hazen, 2013; Kish et al., 2018; McCann et al., 2012). Given the burden on caregivers, official complaints to schools are likely to be underreported. We recommend further research on caregivers’ and schools’ engagement in processes (e.g. monitoring, complaint procedures) to ensure that pupils with medical conditions are justly supported.

The use of candidacy theory strengthened the analysis by enabling us to explain why participants found accessing care to be so challenging, and connects the study to other literature on healthcare access that shows access is about more than the supply of services (Chinn & Abraham, 2016; Fisher et al., 2024; Nkosi et al., 2019). We did not find evidence of ‘identification’, ‘appearing at services’, and ‘offers of, resistance to services’, perhaps due to self-selection – participants had already self-identified and articulated their needs by taking part in the online survey, or because our survey questions were not informed by the theory. Other studies have identified a lack of parental knowledge (i.e. identification of candidacy by parents) as a barrier to support (Sanford et al., 2020; Stavrou Stavros & Demetriou Loucia, 2021; Uhm et al., 2020).

### Strengths and Limitations

This study was able to triangulate the experiences of multiple informants to identify common experiences in negotiating access across a wide range of different health conditions. There was a large and diverse sample of caregivers, enabling us to see a saturation of themes within their sample. However, we did not achieve data saturation with the young people and school staff samples, for which we experienced challenges in recruitment. There was high missingness for gender in our survey of young people and caregivers. We used an open-ended text response for gender to encourage inclusivity but, unexpectedly, this was off-putting for some participants.

We did not explore health inequalities within this study, whether people’s access support and experiences of health and wellbeing support differed by their social characteristics (Williams et al., 2022). Further research could explore whether young people and parents perceived that they had been treated differently as a result of their ethnicity, socio-economic status or other social groupings (Bambra, 2022; Coelho et al., 2022) or compare differences in experiences between these groups (which we were not able to do due to our small sample size). We also recommend further research on the school experiences of young people and caregivers with English as a second language, with poorer literacy, or who feel more disengaged from education, who are more likely to experience disadvantage in accessing support.

## Conclusion

We make several policy and practice recommendations. The health and wellbeing support options that young people with chronic health conditions can be offered should be clarified in government guidance. A named individual to co-ordinate support could strengthen communication across multiple parties regarding a pupil’s health and educational needs, the support options the school can feasibly provide, and if needed, advice on additional sources of support (e.g., from the healthcare provider, charities, private providers, the local authority). Children with health-related absences should be recognised for efforts to engage with learning. Further research is needed on the prevalence and utility of IHPs, and caregiver and school engagement in procedures to ensure that pupils with medical conditions are justly supported.

## Additional Files

The additional files for this article can be found as follows:

10.5334/cie.149.s1 Supplementary File 1. Additional methodological details.

10.5334/cie.149.s2 Supplementary File 2. List of organisations contacted for survey recruitment.

10.5334/cie.149.s3 Supplementary File 3. Survey questions.

10.5334/cie.149.s4 Supplementary File 4. Conditions reported by young people and caregivers.

10.5334/cie.149.s5 Supplementary File 5. Reasonable adjustments described by participants.
