# Supplementary material for: Negotiating Access to Health and Wellbeing Support in Schools for Young People with Chronic Health Conditions in English Secondary Schools: A Qualitative Multi-Informant Study
Source: Contin Educ. 2025 Feb 17;6(1):22–37. doi: 10.5334/cie.149 (PMC11843927; doi:10.5334/cie.149)
Supplement: Supplementary File 1. — Additional methodological details. [file cie-6-1-149-s1.pdf]

Negotiating access to health and wellbeing support in schools for young people with chronic health conditions in English secondary schools: a qualitative multi-informant study

Supplementary File 1: Additional methodological details

Herlitz, L., Jay, M. A., Powell, C., Gilbert, R. & Blackburn, R.

Definitions

Chronic health conditions: Young people and caregivers were eligible to participate if a young person reported that they had one or more conditions (physical or mental) which required healthcare input for one year or more, regardless of whether a young person had been given a diagnosis.

Access: Using literature focused on patients' access to healthcare services, we conceptualised access as more than individuals reaching and taking up the support or services supplied, and defined it as opportunities for pupils, caregivers and staff to identify a pupil's health and wellbeing needs, to seek, reach and use support services, and to have their health and wellbeing needs met (Gulliford et al. 2002; Levesque, Harris, and Russell 2013).

Health and wellbeing support: We defined support as: emotional support from staff; resources, including access to specialist staff and additional staff time; healthcare plans; and reasonable adjustments to ensure equity with peers, including adjustments related to catching up and keeping up with academic work. Falling behind as a result of health-related absences has been reported a significant source of anxiety for pupils with medical conditions (Spencer et al. 2023).

Individual healthcare plan (IHP): An IHP is a non-statutory document that a school can create in partnership with healthcare professionals, caregivers and YP to summarise a pupil's needs, keep a record of contact details for key people, and clarify the actions/expectation of different parties. An IHP can be put into place as soon as a health need is identified and does not depend on a formal statutory process. The school is responsible for ensuring that a plan is finalised and implemented, though healthcare professionals may take the lead on writing it (Department for Education 2015). Templates for IHPs can be found in Department for Education guidance and from the Health Conditions in Schools Alliance website (Department for Education 2015; HCSA n.d.)

Education Health and Care Plan (EHCP): An EHCP is distinct from an IHP. It is a legal document for a young person with special education needs and disabilities whose needs cannot be met by mainstream educational provision within their existing resources. Parents must apply to the local authority for an Education Health and Care assessment. Around 4.3% of all school pupils in England have an EHCP (Zylbersztejn et al. 2023).

Consultation with young people and parents

We consulted with a group of parents of young people with chronic health conditions through the National Children's Bureau's (NCB) in May 2022 about their experiences and research priorities. Their feedback informed our decision to adopt an inclusive definition of chronic health condition (see 2.2), and our survey questions. We sense-checked and discussed the emerging findings with the NCB's Young Research Advisors' group and the parent group in June and September 2023 respectively. Parents highlighted the challenges they had in communicating with the school, identifying who held

This document contains supplementary material for the above-mentioned article, as provided by the authors.
The original article can be downloaded from https://doi.org/10.5334/cie.149

responsibility for school support, accessing tailored support, and challenging schools and local authorities when inadequate support was provided.

Sampling and recruitment

We included young people aged 16+ years only so that they could reflect on their full school experience and give informed consent. We aimed to recruit 30 young people and 30 caregivers with a wide range of conditions to achieve a 'medium-size' qualitative sample (Braun et al. 2021). We aimed to recruit 35 secondary school staff with a diversity of roles.

Data collection

Ethnicity groups were based on UK Office for National Statistics categories, with the exception of adopting two separate categories for White British and White European or Other. These categories were separated based on our consultation with a young person from a mental health charity participation group.

Young people and caregivers were given the option to contact the research team if they preferred to take part by email or Whatsapp voice messages; no requests were received.

References

Braun, Virginia, Victoria Clarke, Elicia Boulton, Louise Davey, and Charlotte McEvoy. 2021. 'The Online Survey as a *Qualitative* Research Tool'. *International Journal of Social Research Methodology* 24 (6): 641–54. https://doi.org/10.1080/13645579.2020.1805550.

Department for Education. 2015. *Supporting Pupils at School with Medical Conditions: Statutory Guidance for Governing Bodies of Maintained Schools and Proprietors of Academies in England*. London: Department for Education. https://assets.publishing.service.gov.uk/government/uploads/system/uploads/attachment_data/file/803956/supporting-pupils-at-school-with-medical-conditions.pdf.

Gulliford, Martin, Jose Figueroa-Munoz, Myfanwy Morgan, David Hughes, Barry Gibson, Roger Beech, and Meryl Hudson. 2002. 'What Does "access to Health Care" Mean?' *Journal of Health Services Research & Policy* 7 (3): 186–88. https://doi.org/10.1258/135581902760082517.

HCSA. n.d. 'Individual Healthcare Plan (IHP)'. http://www.medicalconditionsatschool.org.uk/.

Levesque, Jean-Frederic, Mark F Harris, and Grant Russell. 2013. 'Patient-Centred Access to Health Care: Conceptualising Access at the Interface of Health Systems and Populations'. *International Journal for Equity in Health* 12 (1): 18. https://doi.org/10.1186/1475-9276-12-18.

Spencer, Bethan K C, Judy Wright, Kate Flemming, David Cottrell, and Simon Pini. 2023. 'School Lives of Adolescent School Students Living with Chronic Physical Health Conditions: A Qualitative Evidence Synthesis'. *Archives of Disease in Childhood* 108 (3): 225–29. https://doi.org/10.1136/archdischild-2022-324874.

Zylbersztejn, Ania, Kate Lewis, Vincent Nguyen, Jacob Matthews, Isaac Winterburn, Lucy Karwatowska, Sarah Barnes, et al. 2023. 'Evaluation of Variation in Special Educational Needs Provision and Its Impact on Health and Education Using Administrative Records for England: Umbrella Protocol for a Mixed-Methods Research Programme'. *BMJ Open* 13 (11): e072531. https://doi.org/10.1136/bmjopen-2023-072531.

This document contains supplementary material for the above-mentioned article, as provided by the authors.

The original article can be downloaded from https://doi.org/10.5334/cie.149
