# Supplementary material for: Negotiating Access to Health and Wellbeing Support in Schools for Young People with Chronic Health Conditions in English Secondary Schools: A Qualitative Multi-Informant Study
Source: Contin Educ. 2025 Feb 17;6(1):22–37. doi: 10.5334/cie.149 (PMC11843927; doi:10.5334/cie.149)
Supplement: Supplementary File 2. — List of organisations contacted for survey recruitment. [file cie-6-1-149-s2.pdf]

Negotiating access to health and wellbeing support in schools for young people with chronic health conditions in English secondary schools: a qualitative multi-informant study

Supplementary File 2: List of organisations contacted for survey recruitment

Herlitz, L., Jay, M. A., Powell, C., Gilbert, R. & Blackburn, R.

**Organisations**
Action For ME
Anaphylaxis UK
Anna Freud Centre
Anna Freud Schools in Mind
Association for Young People's Health
Asthma and Lung UK (Respiratory Voices Network)
CCAA – Kids With Arthritis
Children & Young People's Mental Health Coalition
Children's Heart Federation
Children's Society (Young Trustees)
Cystic Fibrosis Trust
Diabetes UK
Epilepsy Action
Great Ormond Street Hospital PPI group
Health Conditions in Schools Alliance
Healthwatch Blackpool (Young Healthwatch)
Healthwatch Bradford (Young Healthwatch)
Healthwatch Brent (Young Healthwatch)
Healthwatch Brighton&Hove (Young Healthwatch)
Healthwatch Central West London (Young Healthwatch)
Healthwatch Dudley (Young Healthwatch)
Healthwatch Gateshead (Young Healthwatch)
Healthwatch Gloucestershire (Young Healthwatch)
Healthwatch Kingston (Young Healthwatch)
Healthwatch Leeds (Young Healthwatch)
Healthwatch Manchester (Young Healthwatch)
Healthwatch Newcastle (Young Healthwatch)
Healthwatch Newham (Young Healthwatch)
Healthwatch North East Lincolnshire (Young Healthwatch)
Healthwatch Northamptonshire (Young Healthwatch)
Healthwatch Sandwell (Young Healthwatch)
Healthwatch Sheffield (Young Healthwatch)
Healthwatch Solihull (Young Healthwatch)
Healthwatch Wakefield (Young Healthwatch)
Healthwatch Walsall (Young Healthwatch)
Healthwatch Wiltshire (Young Healthwatch)
Kidney Care UK
Mental Health Foundation

This document contains supplementary material for the above-mentioned article, as provided by the authors.
The original article can be downloaded from https://doi.org/10.5334/cie.149

RCPCH &Us Engagement Team
Research Consortium for School Health & Nutrition
School Nursing ABUHB
Sickle Cell & Young Stroke Survivors
Sickle Cell Society
Teenage Cancer Trust (Youth Advisory Group)
The Migraine Trust
UCL Institute of Child Health
Versus Arthritis
Well at School
Young Epilepsy (Young reps)
Young Healthwatch Rutland
Young Lives Vs Cancer
YoungMinds
Youthwatch Darlington
Youthwatch Stockport
Youthwatch Trafford
Youth Access

This document contains supplementary material for the above-mentioned article, as provided by the authors.
The original article can be downloaded from https://doi.org/10.5334/cie.149
