# Supplementary material for: Negotiating Access to Health and Wellbeing Support in Schools for Young People with Chronic Health Conditions in English Secondary Schools: A Qualitative Multi-Informant Study
Source: Contin Educ. 2025 Feb 17;6(1):22–37. doi: 10.5334/cie.149 (PMC11843927; doi:10.5334/cie.149)
Supplement: Supplementary File 3. — Survey questions. [file cie-6-1-149-s3.pdf]

# Negotiating access to health and wellbeing support in schools for young people with chronic health conditions in English secondary schools: a qualitative multi-informant study

## *Supplementary File 3: Survey questions*

Herlitz, L., Jay, M. A., Powell, C., Gilbert, R. & Blackburn, R.

### Young person and parent/carer survey

| On-screen page | Onscreen text |
|---|---|
| 1 | Hello!<br><br>Thank you for your interest in our research study. We'd like to explore **the links between chronic health conditions, learning and time off from school** from the perspective of young people, parents and carers, and school staff.<br><br>For us, 'chronic health conditions' mean any **physical** or **mental** health conditions that need **a year or more** of health care support - this might be from a GP, nurse, psychologist or another medical professional, regardless of whether the appointments are regular or not.<br><br>You might have a diagnosed condition (for example, diabetes, asthma, or anxiety) or you might not have a diagnosis. Your condition might be serious or mild.<br><br>Please click on this information sheet for more details about the study.<br><br>To take part in this survey, you must be based in the UK and one of the following people:<br><br>1) **A young person** between 16 and 25 years old who attended a mainstream secondary school for all or most of your school years and had one or more chronic health conditions. You may still have ongoing health problems now.<br><br>2) **A parent or carer** of a child aged between 11 and 25 years who attends (or attended) a mainstream secondary school for all or most of their school years and has (or had) one or more chronic health conditions.<br><br>If you're interested in taking part, please click 'next page' below. |
| 2 | You can complete the survey on your phone or computer. The survey involves giving a few details about yourself (e.g. age) and answering five or six questions about your views. You don't need to share anything that you don't want to. One of the questions is related to another study that you can choose to answer if you wish – we will highlight when this is being asked.<br><br>The survey will take about 20 minutes to complete and you don't have to do it all in one go. Please be aware that if you are using a shared phone or computer and want to leave |

This document contains supplementary material for the above-mentioned article, as provided by the authors.

The original article can be downloaded from https://doi.org/10.5334/cie.149

| | | |
|---|---|---|
| | the survey in the middle of it and come back to it later, someone else could see your answers if you do not lock your phone or computer. You will need an internet connection to complete the survey. | |
| 3 | We are offering a prize draw to thank participants for the time they are giving. We have £50 Love-to-shop e-vouchers to give away to six young people and six parents/carers. We hope that 30 young people and 30 parents/carers will complete the survey. If every one of those people enter the prize draw, every person would have a 1 in 5 chance of winning £50.<br><br>You can complete the survey anonymously. However, if you would like to enter the prize draw or be involved in future research, you will need to give your name and email address at the end of the survey and you will no longer be anonymous to the research team. Your details will be stored separately from your survey answers, and the survey will contain no identifying information when it is analysed and written up in study reports. Your name and email address will be deleted once the prize draw has taken place on 28th February 2023. | |
| 4 | If you have any questions or have problems completing the survey, please email [name of researcher] at [email of researcher] who'll be happy to help you. If you find the survey format difficult, let [name of researcher] know and we can offer the survey by email or Whatsapp for the first few people who need it.<br><br>If you're happy to take part, click on the arrow to go through our consent questions. | |
| 5 | Consent form – see appendix 3 | |
| 6 | Please tick the answer that applies to you.<br>o I am a young person who has (or had) chronic health problems.<br>o I am a parent or carer of a young person who has (or had) chronic health problems. | |
| 7 | **Chronic health conditions and school**<br><br>We're not interested in spelling or grammar! Please use emojis if that's helpful.<br><br>There are no right or wrong answers, we'd like to know your ideas so there's no limit to the number of words you can use. In fact, the more you can tell us, the better!<br><br>As a reminder, we describe 'chronic health conditions' as any **physical** or **mental** health problem that needs support from a doctor, nurse, psychologist or another medical professional **for a year or more**. A young person might have a diagnosed condition, they might have more than one condition, or they might not have a diagnosis. The condition might be serious or mild. | |
| | *Survey filtered hereafter for YP based on p6 answer.* | *Survey filtered hereafter for parent or carer based on p6 answer.*<br><br>How many children aged 11 – 25 years do you care for with chronic health conditions? |
| | | If you have more than one child of secondary-school age with a chronic health condition, please choose one child about whom to answer the rest of the survey questions. |

This document contains supplementary material for the above-mentioned article, as provided by the authors.

The original article can be downloaded from https://doi.org/10.5334/cie.149

| | | |
|---|---|---|
| | In your own words, please describe all the chronic health conditions you have (or had) during your time at secondary school and any diagnoses. | Please describe all the chronic health conditions your child has (or had) during their time at secondary school, and any diagnoses. |
| | Did you have an Individual Health Care Plan when you were at secondary school?<br><br>o Yes<br>o No<br>o I don’t know | Does/did your child have an Individual Health Care Plan during their time at secondary school?<br><br>o Yes<br>o No<br>o I don’t know |
| | Do you have any special educational needs?<br><br>o Yes<br>o No<br>o I don’t know | Does your child have any special educational needs?<br><br>o Yes<br>o No<br>o I don’t know |
| | 1) In your own words, please describe how you feel chronic health conditions have affected your time at secondary school.<br><br>You might want to think about things like:<br>a. Attendance and taking time off<br>b. Relationships with friends<br>c. Taking exams and completing work<br>d. Anything else you want to tell us | 1) In your own words, please describe how you feel chronic health conditions have affected your child’s time at secondary school.<br><br>You might want to think about things like:<br>a. Attendance and taking time off<br>b. Relationships with friends<br>c. Taking exams and completing work<br>d. Anything else you want to tell us |
| | 2) During secondary school, was there anything really supportive that someone did so that you could do your best?<br><br>For example, a friend, a particular teacher, head of year, SENCO, a family member.<br><br>If nobody did anything supportive, was there anything someone could have done to make things easier? | 2) During secondary school, was there anything really supportive that someone did so that your child could do their best?<br><br>For example, a friend, a particular teacher, head of year, SENCO, a family member.<br><br>If nobody did anything supportive, was there anything someone could have done to make things easier? |
| | 3) What do you think are the most difficult things facing young people with chronic health conditions in schools? | 3) What do you think are the most difficult things facing young people with chronic health conditions in schools? |
| | 4) If you could change anything in schools or related to healthcare services that would have made your time in school easier, what would you do and why? | 4) If you could change anything in schools or related to healthcare services that would have made your child’s time in school easier, what would you do and why? |
| | *This question was wrongly filtered and did <u>not</u> appear in the final YP survey.* | 5) How did carrying out school work at home during the COVID-19 pandemic |

This document contains supplementary material for the above-mentioned article, as provided by the authors.
The original article can be downloaded from https://doi.org/10.5334/cie.149

| | | |
|---|---|---|
| | 5) How did carrying out school work at home during the COVID-19 pandemic in 2020-21 affect your participation in school? | in 2020-21 affect your child's participation in school?<br><br>Leave blank if your child had finished school before the pandemic. |
| | **Demographic questions**<br><br>We are collecting a little bit of information about our participants so that we can describe who took part (without identifying you). We are using Office for National Statistics (ONS) categories. Please select the category that best applies to you or fill in where necessary. If you prefer not to answer any questions, leave them blank. | **Demographic questions**<br><br>We are collecting a little bit of information about our participants so that we can describe who took part (without identifying you). We are using Office for National Statistics (ONS) categories. Please select the category that best applies to you or fill in where necessary. If you prefer not to answer any questions, leave them blank. |
| | Please describe your gender in your own words (or if you prefer, use one of these categories – female, male, trans, non-binary). | Please describe your gender in your own words (or if you prefer, use one of these categories – female, male, trans, non-binary). |
| | How old are you?<br><br>○ 16 to 18 years<br>○ 19 to 25 years | How old are you?<br><br>○ 26 to 35 years<br>○ 36 to 45 years<br>○ 46 to 55 years<br>○ 56 to 65 years<br>○ 66+ years |
| | What is your ethnic background?<br><br>○ Asian or Asian British (Indian, Pakistani, Bangladeshi, Chinese, Any other Asian background)<br>○ Black, Black British, Caribbean or African (Caribbean, African, Any other Black, Black British or Caribbean background)<br>○ Mixed or Multiple ethnic groups (White and Black Caribbean, White and Black African, White and Asian, Any other Mixed or multiple ethnic background)<br>○ White British (English, Welsh, Scottish, Northern Irish or British, Irish, Gypsy or Irish Traveller, Roma)<br>○ White European or Other (Any other White background)<br>○ Other ethnic group (Arab, any other ethnic group)<br>○ Prefer not to say | What is your ethnic background?<br><br>○ Asian or Asian British (Indian, Pakistani, Bangladeshi, Chinese, Any other Asian background)<br>○ Black, Black British, Caribbean or African (Caribbean, African, Any other Black, Black British or Caribbean background)<br>○ Mixed or Multiple ethnic groups (White and Black Caribbean, White and Black African, White and Asian, Any other Mixed or multiple ethnic background)<br>○ White British (English, Welsh, Scottish, Northern Irish or British, Irish, Gypsy or Irish Traveller, Roma)<br>○ White European or Other (Any other White background)<br>○ Other ethnic group (Arab, any other ethnic group)<br>○ Prefer not to say |

This document contains supplementary material for the above-mentioned article, as provided by the authors.

The original article can be downloaded from https://doi.org/10.5334/cie.149

<table>
<tr><td></td><td></td><td></td></tr>
<tr><td></td><td>Which part of the UK are you based in?<br><br>○ North East England (for example, Darlington, Durham, Newcastle-upon-Tyne)<br>○ North West England (for example, Blackpool, Liverpool, Manchester)<br>○ Yorkshire and the Humber (for example, Leeds, York, Sheffield)<br>○ East Midlands (for example, Derby, Leicester, Nottingham)<br>○ West Midlands (for example, Birmingham, Coventry, Wolverhampton)<br>○ East of England (for example, Bedford, Cambridge, Essex)<br>○ London<br>○ South East (for example, Brighton, Kent, Reading)<br>○ South West (for example, Bristol, Devon, Cornwall)</td><td>Which part of the UK are you based in?<br><br>○ North East England (for example, Darlington, Durham, Newcastle-upon-Tyne)<br>○ North West England (for example, Blackpool, Liverpool, Manchester)<br>○ Yorkshire and the Humber (for example, Leeds, York, Sheffield)<br>○ East Midlands (for example, Derby, Leicester, Nottingham)<br>○ West Midlands (for example, Birmingham, Coventry, Wolverhampton)<br>○ East of England (for example, Bedford, Cambridge, Essex)<br>○ London<br>○ South East (for example, Brighton, Kent, Reading)<br>○ South West (for example, Bristol, Devon, Cornwall)</td></tr>
<tr><td></td><td>Thank you for answering the survey questions. Would you like to be entered into the prize draw for a one in five chance to win a £50 love2shop e-voucher?<br><br>○ Yes<br>○ No</td><td>Thank you for answering the survey questions. Would you like to be entered into the prize draw for a one in five chance to win a £50 love2shop e-voucher?<br><br>○ Yes<br>○ No</td></tr>
<tr><td></td><td>(If answered yes)<br>Please provide your name and email address and we will enter your details into the prize draw.</td><td>(If answered yes)<br>Please provide your name and email address and we will enter your details into the prize draw.</td></tr>
<tr><td></td><td colspan="2">Thank you for taking part in this survey. Your responses have been submitted.<br><br>We appreciate the time you have taken to answer these questions and we know that thinking about negative experiences can be stressful. We hope the findings will be useful in improving policy and practice for young people with long term health conditions. If you have any questions or feedback, please email [name of researcher]: [email of researcher]<br><br>If you gave us your email address for the prize draw and/or to take part in future research, we will delete this from your survey responses within 3 days and save it separately in a secure database.<br><br>Below are some specialist organisations if you need any further support or information related to chronic health conditions and schooling:<br>• https://www.childline.org.uk/get-support/ tel: 0800 1111 a 24-hours, 7-days-a-week phone line for young people up to 19 years for help and advice.<br>• http://www.medicalconditionsatschool.org.uk/ An alliance of charities supporting children and young people in school</td></tr>
</table>

This document contains supplementary material for the above-mentioned article, as provided by the authors.

The original article can be downloaded from https://doi.org/10.5334/cie.149

| | |
|---|---|
| | • https://www.youngminds.org.uk/A national young people's mental health charity<br>• https://stateofchildhealth.rcpch.ac.uk/evidence/long-term-conditions/ Royal College of Paediatrics and Child Health RCPCH_ evidence review of long-term conditions. RCPCH is the membership organisation for paediatricians in the UK. |

## School staff survey

| Onscreen page | Onscreen text |
|---|---|
| 1 | Hello!<br><br>Thank you for your interest in our study. We'd like to explore **the links between chronic health conditions, learning and time off from school** from the perspective of school staff, young people, and parents and carers.<br><br>For us, 'chronic health conditions' mean any **physical** or **mental** health conditions that need **a year or more** of health care support - this might be from a GP, nurse, psychologist or another medical professional, regardless of whether the appointments are regular or not.<br><br>A young person might have a diagnosed condition (for example, diabetes, asthma, or anxiety) or they might not have a diagnosis (for example, experiencing severe fatigue). Their condition might be serious or mild.<br><br>Please click on this information sheet for more details about the study.<br><br>To take part in this survey, you must be based **in England** and work in a **mainstream secondary school**. You will have one of the following roles:<br><br>1) A member of staff that teaches or supports students e.g. classroom teacher, teaching assistant, SENCO.<br><br>2) A member of staff with a leadership role and/or responsibilities for writing school policies, e.g. Head of Year, Head of Inclusion, Assistant or Deputy Headteacher, Headteacher. You may also have teaching responsibilities.<br><br>3) An educational psychologist.<br><br>If you're interested in taking part, please click 'next page' below. |
| 2 | You can complete the survey on your phone or computer. The survey involves answering **four** open-ended questions about your views and giving a few details about yourself (e.g. age). You don't need to share anything that you don't want to.<br><br>The survey will take about **10 minutes** to complete. Please be aware that if you are using a shared phone or computer and want to leave the survey in the middle of it and come back to it later, someone else could see your answers if you do not lock |

This document contains supplementary material for the above-mentioned article, as provided by the authors.
The original article can be downloaded from https://doi.org/10.5334/cie.149

<table>
<tr><td></td><td>your phone or computer. You will need an internet connection to complete the survey.</td></tr>
<tr><td>3</td><td>We are offering a prize draw to thank participants for the time they are giving. We have £50 Love-to-shop e-vouchers to give away to seven school staff. We hope that 35 school staff will complete the survey. If every one of those people enter the prize draw, every person would have a 1 in 5 chance of winning £50.<br><br>You can complete the survey anonymously. However, if you would like to enter the prize draw, you will need to give your name and email address at the end of the survey and you will no longer be anonymous to the research team. Your details will be stored separately from your survey answers, and the survey will contain no identifying information when it is analysed and written up in study reports. Your name and email address will be deleted once the prize draw has taken place on 13th March 2023.</td></tr>
<tr><td>4</td><td>If you have any questions or have problems completing the survey, please email [name of researcher] at [email of researcher] who’ll be happy to help you.<br><br>If you’re happy to take part, click on the arrow to go through our consent questions.</td></tr>
<tr><td>5</td><td>Consent form – see appendix 3</td></tr>
<tr><td>6</td><td>Please tick the answer that applies to you.<br><br>o I am a member of staff teaching or supporting students e.g. classroom teacher, teaching assistant, SENCO. I do not have a leadership role or responsibilities for writing school policies.<br>o I am a member of staff with a leadership role and/or responsibilities for writing school policies e.g. head of year, head of inclusion, assistant or deputy head teacher, head of year, head of department.<br>o I am an educational psychologist.</td></tr>
<tr><td>7</td><td>Chronic health conditions and school<br><br>There are no right or wrong answers, we’d like to know your ideas so there’s no limit to the number of words you can use. In fact, the more you can tell us, the better!<br><br>As a reminder, we describe ‘chronic health conditions’ as<br>any physical or mental health problem that needs support from a doctor, nurse, psychologist or another medical professional for a year or more. A young person might have a diagnosed condition, they might have more than one condition, or they might not have a diagnosis. The condition might be serious or mild.</td></tr>
<tr><td>8</td><td>1) Please describe an example of when a student with a chronic physical and/or mental health condition was particularly well supported during their time at your school. This may have been something you did or something you observed in your place of work.</td></tr>
<tr><td>9</td><td>2) Are there any groups of students with health conditions that are harder to support than others? If so, why?<br>You might want to think about challenges associated with particular:<br>• Types of health conditions<br>• Student or family circumstances<br>• Diagnosis or treatment stage</td></tr>
<tr><td>10</td><td>3) Based on your experience, what are the main challenges facing school staff in providing effective support for young people with chronic health conditions?</td></tr>
</table>

This document contains supplementary material for the above-mentioned article, as provided by the authors.
The original article can be downloaded from https://doi.org/10.5334/cie.149

| | |
|---|---|
| 11 | 4) If you could change one thing right now about the school you work in, or education or healthcare services more broadly, to make it easier for young people with chronic health conditions to learn and achieve, what would it be and why? |
| 12 | **Demographic questions**<br><br>We are collecting a little bit of information about our participants so that we can describe who took part (without identifying you). We are using Office for National Statistics (ONS) categories. Please select the category that best applies to you or fill in where necessary. If you prefer not to answer any questions, leave them blank. |
| 13 | Please describe your gender in your own words (or if you prefer, use one of these categories – female, male, trans, non-binary). |
| 14 | How old are you?<br><br>○ 19 to 25 years<br>○ 26 to 35 years<br>○ 36 to 45 years<br>○ 46 to 55 years<br>○ 56 to 65 years<br>○ 66+ years |
| 15 | What is your ethnic background?<br><br>○ Asian or Asian British (Indian, Pakistani, Bangladeshi, Chinese, Any other Asian background)<br>○ Black, Black British, Caribbean or African (Caribbean, African, Any other Black, Black British or Caribbean background)<br>○ Mixed or Multiple ethnic groups (White and Black Caribbean, White and Black African, White and Asian, Any other Mixed or multiple ethnic background)<br>○ White British (English, Welsh, Scottish, Northern Irish or British, Irish, Gypsy or Irish Traveller, Roma)<br>○ White European or Other (Any other White background)<br>○ Other ethnic group (Arab, any other ethnic group)<br>○ Prefer not to say |
| 16 | Which part of the UK are you based in?<br><br>○ North East England (for example, Darlington, Durham, Newcastle-upon-Tyne)<br>○ North West England (for example, Blackpool, Liverpool, Manchester)<br>○ Yorkshire and the Humber (for example, Leeds, York, Sheffield)<br>○ East Midlands (for example, Derby, Leicester, Nottingham)<br>○ West Midlands (for example, Birmingham, Coventry, Wolverhampton)<br>○ East of England (for example, Bedford, Cambridge, Essex)<br>○ London<br>○ South East (for example, Brighton, Kent, Reading)<br>○ South West (for example, Bristol, Devon, Cornwall) |
| 17 | Please describe your current role in secondary schools? |
| 18 | How long have you been working as a teacher, member of school staff, or educational psychologist? |

This document contains supplementary material for the above-mentioned article, as provided by the authors.
The original article can be downloaded from https://doi.org/10.5334/cie.149

| | |
|---|---|
| | ○ Less than a year<br>○ 1 – 2 years<br>○ 3 – 5 years<br>○ 6 – 9 years<br>○ 10 years or more |
| 19 | Approximately how many students are taught at your school?<br><br>○ Less than 500 students<br>○ 500 to 899 students<br>○ 900 to 1199 students<br>○ 1200 or more students |
| 20 | Thank you for answering the survey questions. Would you like to be entered into the prize draw for a one in five chance to win a £50 love2shop e-voucher?<br><br>○ Yes<br>○ No |
| 21 | (If answered yes)<br>Please provide your name and email address and we will enter your details into the prize draw. |
| 22 | Thank you for taking part in this survey. Your responses have been submitted.<br><br>We appreciate the time you have taken to answer these questions. We hope the findings will be useful in improving policy and practice for young people with long term health conditions. If you have any questions or feedback, please email [name of researcher]: [email of researcher].<br><br>If you gave us your email address for the prize draw and/or to take part in future research, we will delete this from your survey responses within 3 days and save it separately in a secure database.<br><br>Below are some specialist organisations if you need any further support or information related to chronic health conditions and schooling:<br>• http://www.medicalconditionsatschool.org.uk/ An alliance of charities supporting children and young people in school<br>• https://www.youngminds.org.uk/A national young people's mental health charity<br>• https://stateofchildhealth.rcpch.ac.uk/evidence/long-term-conditions/ Royal College of Paediatrics and Child Health RCPCH - evidence review of long-term conditions. RCPCH is the membership organisation for paediatricians in the UK. |

This document contains supplementary material for the above-mentioned article, as provided by the authors.
The original article can be downloaded from https://doi.org/10.5334/cie.149
