# Supplementary material for: Negotiating Access to Health and Wellbeing Support in Schools for Young People with Chronic Health Conditions in English Secondary Schools: A Qualitative Multi-Informant Study
Source: Contin Educ. 2025 Feb 17;6(1):22–37. doi: 10.5334/cie.149 (PMC11843927; doi:10.5334/cie.149)
Supplement: Supplementary File 4. — Conditions reported by young people and caregivers. [file cie-6-1-149-s4.pdf]

Negotiating access to health and wellbeing support in schools for young people with chronic health conditions in English secondary schools: a qualitative multi-informant study

Supplementary File 4: Conditions reported by young people and caregivers

Herlitz, L., Jay, M. A., Powell, C., Gilbert, R. & Blackburn, R.

**Name of condition reported by participant**	**Number of YP/caregivers reporting condition**
Anxiety	13
Myalgic encephalomyelitis (ME)/chronic fatigue syndrome (CFS)	9
Autism	7
Asthma	7
Depression	6
Attention Deficit and Hyperactivity Disorder (ADHD)	4
Delayed Sleep Phase disorder (DSPS) or other sleep disorders	4
Hypermobile Ehlers-Danlos Syndrome (HEDS) or Hybermobility	4
Epilepsy	3
Postural tachycardia syndrome (PoTS)	3
Sickle cell anaemia	3
Obsessive Compulsive Disorder	2
Post Traumatic Stress Disorder (PTSD)	2
Sensory processing dysfunction (SPD)	2
Developmental Co-ordination Disorder (DCD)	2
Type 1 diabetes	2
Avoidant restricted food intake disorder (ARFID)	1
Bulimia	1
Developmental Trauma	1
Aspergers Syndrome	1
Learning disability	1
Cavernoma	1
Motor and verbal tics	1
Neurofibromatosis Type 1 (NF1)	1
New Daily Chronic Headaches	1
Right sided hemiplegia	1
Allergies	1
Anaphylaxis	1
Arterial switch heart operation at birth	1
Bronchiectasis	1
Complex Gut Disease	1
Complex Regional Pain Syndrome	1
Congenital Adrenal Hyperplasia	1
Constant chest infections and tonsilitis	1
Cystic fibrosis	1
Cystic hygroma	1

This document contains supplementary material for the above-mentioned article, as provided by the authors.
The original article can be downloaded from https://doi.org/10.5334/cie.149

Dilated cardiomyopathy	1
Gastroparesis	1
Gastrostomy	1
Inappropriate Sinus Tachycardia (IST)	1
Inflammatory anaemia	1

This document contains supplementary material for the above-mentioned article, as provided by the authors.
The original article can be downloaded from https://doi.org/10.5334/cie.149
