# Supplementary material for: Negotiating Access to Health and Wellbeing Support in Schools for Young People with Chronic Health Conditions in English Secondary Schools: A Qualitative Multi-Informant Study
Source: Contin Educ. 2025 Feb 17;6(1):22–37. doi: 10.5334/cie.149 (PMC11843927; doi:10.5334/cie.149)
Supplement: Supplementary File 5. — Reasonable adjustments described by participants. [file cie-6-1-149-s5.pdf]

Negotiating access to health and wellbeing support in schools for young people with chronic health conditions in English secondary schools: a qualitative multi-informant study

Supplementary File 5: Reasonable adjustments described by participants

Herlitz, L., Jay, M. A., Powell, C., Gilbert, R. & Blackburn, R.

Access to resources to support education during and after school absences

- Access to an online classroom
- Providing iPads for remote work
- Being given information about work missed

Adjustments to manage the duration of the school day

- Reduced or bespoke timetable
- Access to a separate room or pastoral room for respite or a change in learning environment
- Lunch plans

Adjustments to school rules

- Flexible rules on uniform (e.g. being allowed to untuck shirt for insulin pump)

Access to other spaces within the school

- Lift pass
- Toilet pass/care or use of an adult disabled toilet
- Personal evacuation plan
- Using less crowded school entrances and exits

Adjustments within the classroom

- Changing position of child in classroom (e.g. back of the class for more privacy, front of the class for easier exit)
- Equipment for sensory impairment (e.g. materials in Braille, hearing loops)
- Sensory equipment (e.g. ear defenders, using coloured paper)
- Being able to use a mobile phone in lessons (e.g. to support use of insulin pump)
- Use of laptop in class
- Flexibility on staying in the classroom/rest breaks

Adjustments with assessment

- Extensions for coursework or homework
- Flexibility on grading (grading based on projects and oral participation)
- Exam support (spaced out exams, support with completion of medical forms, extra time, separate room)

Adjustments on school trips

- Personal transportation

This document contains supplementary material for the above-mentioned article, as provided by the authors.
The original article can be downloaded from https://doi.org/10.5334/cie.149
